# The Systemic Inflammatory Response Syndrome (SIRS) in acutely hospitalised medical patients: a cohort study

**DOI:** 10.1186/1757-7241-17-67

**Published:** 2009-12-27

**Authors:** Pål Comstedt, Merete Storgaard, Annmarie T Lassen

**Affiliations:** 1 Department of Infectious Diseases, Odense University Hospital, Odense, Denmark; 2 Department of Infectious Diseases, Århus University Hospital, Skejby, Denmark; 3 Institute of Clinical Research, University of Southern Denmark, Odense, Denmark

## Abstract

**Background:**

Sepsis is an infection which has evoked a systemic inflammatory response. Clinically, the Systemic Inflammatory Response Syndrome (SIRS) is identified by two or more symptoms including fever or hypothermia, tachycardia, tachypnoea and change in blood leucocyte count. The relationship between SIRS symptoms and morbidity and mortality in medical emergency ward patients is unknown.

**Methods:**

We conducted a prospective cohort study of the frequency of SIRS and its relationship to sepsis and death among acutely hospitalised medical patients. In 437 consecutive patients, SIRS status, blood pressure, infection and comorbidity on admission was registered together with 28-day mortality.

**Results:**

A hundred and fifty-four patients (35%) had SIRS on admission, 211 patients (48%) had no SIRS, and 72 patients (16%) had insufficient data to evaluate their SIRS status. SIRS patients were 2.2 times more frequently infected, with 66/154 SIRS patients versus 41/211 non-SIRS patients: p < 0.001, relative risk (RR) 2.2 (95% confidence interval (CI) 1.6-3.1), and they had a 6.9 times higher 28-day mortality than non-SIRS patients with 15/154 SIRS patients versus 3/211 non-SIRS patients: p = 0.001, RR 6.9 (95% CI 2.0-23.3). Most of the deaths among patients with SIRS occurred among patients with malignant conditions. Septic shock developed in 4/154 (3%) of the patients with SIRS, whereas this occurred in only one of the 211 patients (0.5%) without SIRS on arrival: p = 0.08, RR 5.5 (95% CI 0.6-48.6).

**Conclusion:**

We found SIRS status on admission to be moderately associated with infection and strongly related to 28-day mortality.

## Background

Sepsis is a systemic inflammatory response to a confirmed or suspected infection. Clinically, the *Systemic Inflammatory Response Syndrome *(SIRS) is the occurrence of at least two of the following criteria: fever >38.0°C or hypothermia <36.0°C, tachycardia >90 beats/minute, tachypnea >20 breaths/minute, leucocytosis >12*10^9^/l or leucopoenia <4*10^9^/l [1,2].

The development from sepsis to septic shock represents a continuum with increasing mortality. The in-hospital/28-day mortality in severe sepsis is 10%-40% and in septic shock it is 30%-60% [3-11]. Early treatment with antibiotic and fluid resuscitation has been found to be strongly related to increased survival, which makes severe sepsis a condition which is important to identify and treat as early as possible [2,12,13].

Although a few studies have evaluated the progress of SIRS among emergency ward patients with suspected infection, most studies of SIRS have focused on patients in intensive care units (ICUs) [8-11,14,15]. The occurrence and usefulness of registered SIRS status among all acute medical patients in an emergency ward is unknown.

The aim of the present study was to describe the relevance of SIRS in predicting morbidity and mortality among patients in a medical emergency ward.

## Materials and methods

### Patient population

All acutely hospitalised medical patients admitted to the medical emergency ward as well as medical patients admitted directly to ICU, Odense University Hospital in a six-week period (3 September to 14 October 2007) were included. Patients transferred from other wards or hospitals were excluded. If patients had more than one admission to the department during the inclusion period, they were included at the first admission and not at the following admissions.

Odense University Hospital serves as a primary hospital for 185,000 people. The medical emergency ward admits adult patients (> age 15 years) with acute medical conditions, with the exception of patients with a suspected acute heart disease or verified diabetes, chronic gastroenterological, haematological or nephrological disease.

There were no interventions related to the study, and all patients received standard care following the ward's guidelines.

### Data collection and categorisation of patients

*SIRS *was defined as fulfilling at least two of the following four criteria: fever >38.0°C or hypothermia <36.0°C, tachycardia >90 beats/minute, tachypnea >20 breaths/minute, leucocytosis >12*10^9^/l or leucopoenia <4*10^9^/l.

The body temperature, heartbeat frequency and respiratory frequency of all patients were registered on arrival by the nurses. The data were collected a few minutes after the patient arrived in the ward. The nurses were aware of the study and were repeatedly reminded to obtain a full set of observations for all patients. Documentation of infection was based on the clinical evaluation within the first two days after arrival, including clinical examinations as well as radiological evaluation, and where infection was suspected by the clinical doctor or indicated by blood, urine and other cultures. Leucocyte count on arrival and information on previous hospitalisation were obtained from the electronic Patient Administrative System of Funen County, and comorbidity was defined as the main discharge diagnoses (if any) during the last six months.

Follow up was performed on day 28 by recording the occurrence of documented infection, treatment in ICU, start of antibiotic treatment, development of sepsis, severe sepsis or septic shock, length of hospital stay, diagnosis on discharge, 28 day mortality and, if possible, the course of mortality. The follow-up registration was made by chart review by one of the authors (PC), with evaluation by a specialist in infectious diseases (MS or AL) if there were any doubts about interpretation or classification. SIRS status was evaluated in a separate setting, but parameters registered on patient arrival were not blinded in the chart review.

Only infection, sepsis, severe sepsis and septic shock occurring within the first two days of the hospital stay were registered in order to exclude conditions acquired in the hospital.

*Infection *was defined as identification of a relevant pathogen by microscopy/culture/polymerase chain reaction, positive serology, pneumonia verified by chest X-ray, infection documented with other imaging techniques, positive urine dip test combined with symptoms of urine tract infection, or as typical clinical symptoms such as erysipelas.

### Sepsis was defined as SIRS plus a documented infection

*Severe sepsis *was defined as sepsis plus at least one of the following (without other comorbidity/therapeutic explanation): Glasgow coma scale ≤ 14; PaO_2 _≤ 9.75 kPa; oxygen saturation ≤ 92%, PaO_2_/FiO_2 _≤ 250;, pH ≤ 7.3; lactate ≥ 2.5 mmol/l; creatinine ≥ 177 μmol/l; 100% increase of creatinine in patients with known kidney disease; oliguria ≤ 30 ml/h in ≥ 3 h or ≤ 0.7 l/24 h, prothrombin time ≤ 0.6; platelets ≤ 100*10^9^/l; bilirubin ≥ 43 μmol/l; paralytic ileus; systolic blood pressure ≤ 90 mm Hg or systolic blood pressure fall ≥ 40 mm Hg from baseline.

*Septic shock *was defined as sepsis plus a systolic blood pressure of ≤ 90 mmHg or systolic blood pressure fall ≥ 40 mmHg from the baseline despite adequate fluid resuscitation or the use of vasopressor agents.

### Analyses

Based on symptoms on arrival, the patients were categorised with SIRS, non-SIRS, and if essential information was missing, as unknown SIRS status. The categorisation was conducted without a knowledge of the result of any outcome variables.

Patients were compared using a chi-squared test for dichotomous variables and a Mann-Whitney test for continuous variables. P values < 0.05 were considered statistically significant. Relative risk was calculated comparing patients with and without SIRS on arrival, with 95% confidence intervals calculated on the basis of the distribution of the counting data.

EpiData version 3.1 was used for data registration and STATA version 8 (STATA Corporation^®^, Texas, USA) for statistical analysis.

### Ethics

In accordance with Danish regulations, the study was approved by the Danish Data Protection Agency.

## Results

During the enrolment period, a total of 643 patients were admitted to the medical ward or directly to the ICU as medical patients. Of these, 206 were transferred from other wards or had previously participated in the study. The remaining 437 consecutive acute medical patients were enrolled in the study.

A hundred and fifty-four of the 437 patients (35%) had SIRS on arrival, 211 patients (48%) did not have SIRS, and 72 (16%) had unknown SIRS status. Patients with unknown SIRS status were younger than patients with known SIRS status (Table 1). Among patients with known SIRS status, patients without SIRS were younger than patients with SIRS on arrival, and fewer had comorbidity (Table 2).

**Table 1:** Basic characteristics - all patients

	Patients with known SIRS status (N = 365)	Unknown SIRS (N = 72)	P-value
Characteristic	N (%) or median (range)	N (%) or median (range)	
Male sex	173 (47%)	40 (56%)	0.21
			
Age (years)	60 (15-96)	50 (15-88)	0.004
			
Comorbidity	135 (37%)	26 (36%)	0.89
Malignancy	26 (7%)	5 (7%)	0.96
Cardiovascular	18 (5%)	1 (1%)	0.18
Pulmonary disease	28 (8%)	3 (4%)	0.29

Basic characteristics among acute medical patients with and without known systemic inflammatory (SIRS) status.

**Table 2:** SIRS or no SIRS on arrival

Variable	Not SIRS (N = 211)	SIRS (N = 154)	P-value
	N (%) or median (range)	N (%) or median (range)	
Male sex	96 (46%)	77 (50%)	0.40
Age (years)	56 (15-92)	62 (15-96)	0.008
			
Comorbidity	69 (33%)	66 (43%)	0.047
Malignancy	11 (5%)	15 (10%)	0.10
Cardiovascular	10 (5%)	8 (5%)	0.84
Pulmonary disease	9 (4%)	19 (12%)	0.004
			
Documented community-acquired infection	41 (19%)	66 (43%)	<0.001
Positive blood cultures	3 (1%)	8 (5%)	0.06
Mortality on day 28	3 (1%)	15 (10)	<0.001

Basic characteristics and outcome among acute medical patients according to systemic inflammatory response (SIRS) on arrival

### Infection and severity of disease

**Figure 1:**
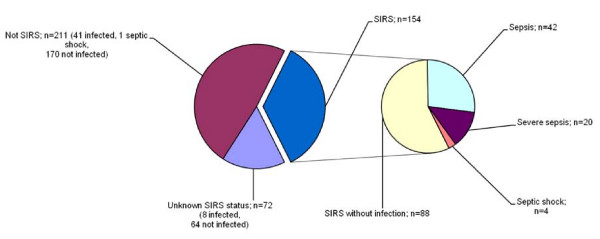
**Acute medical patients according to systemic inflammatory response (SIRS) on arrival, community-acquired infection, sepsis, severe sepsis and septic shock (N = 437)**.

Infection was documented in 66/154 (43%) of the patients with SIRS and in 41/211 (19%) of the non-SIRS patients (p < 0.001) (Figure 1). This corresponds to a 2.2 (95% CI 1.6-3.1) times higher proportion of patients with infection among SIRS patients. Among all 365 patients with known SIRS status, 107 patients had an infection and 66 (62%) presented with SIRS, while 41 (38%) did not.

Septic shock occurred among 4/154 (3%) of the patients with SIRS, while this was only found among one of the 211 patients (0.5%) without SIRS on arrival (p = 0.08). This corresponds to a 5.5 (95% CI 0.6-48.6) times higher proportion of septic shock among patients who have SIRS on arrival compared to patients without SIRS.

Eleven patients had bacteraemia with 8/154 SIRS-positive patients compared to 3/211 patients who did not present with SIRS: p = 0.06, RR 3.7 (95% CI 0.99-13.5).

All patients with SIRS and bacteraemia developed severe sepsis (n = 7) or septic shock (n = 1).

### Mortality

Total mortality at day 28 was 21/437 (5%). Among the 154 patients who presented with SIRS, 15 (10%) died within 28 days compared to 3/211 (1%) non-SIRS patients and 3/72 patients (4%) with unknown SIRS status. This corresponds to a 6.9 (95% CI 2.0-23.3) times higher mortality among SIRS patients than among patients without SIRS (p < 0.001) (Table 2). Among the 15 patients who presented with SIRS and died within 28 days, 13 had no documented infection on arrival, one had sepsis and one had severe sepsis. None of the five patients who presented with septic shock died within 28 days.

## Discussion

We found a high prevalence of SIRS (35%) among acutely hospitalised medical patients, a moderate relation between SIRS and infection (RR 2.2), and a high (10%) 28-day mortality among SIRS patients.

The strength of our study is the consecutive inclusion of all patients from the acute medical emergency ward, the prospective design with identification of symptoms and infection on arrival, and the possibility of following up on all patients with the aid of the unique personal identification numbers used by all Danish citizens in all contact with the hospital system [16]. As all deaths among Danish citizens are registered under their unique personal registration numbers irrespective of whether death occurs in hospital or at home, and as all patients were followed up until death or day 28, whichever came first, we are confident that we have identified all deaths.

The weakness of the study is the fact that not all acute medical patients were admitted to the ward, as patients suspected of suffering acute heart disease or with verified diabetes, chronic gastroenterological, haematological or nephrological disease were admitted directly to other wards. As in other non-blinded observational studies, there is a risk of biased registration which we have tried to avoid by registration of SIRS symptoms and documented infection in two different settings without any knowledge of conclusions from the opposite setting. It is important to remember that the results reflect the standard of care in the actual ward, which is not necessarily generalisable to other wards.

As in other studies [8,15], we found that a high proportion of the acute patients (35%) had SIRS on admission. Other studies have found SIRS to be a predictor of infections, severity of disease, organ failure and outcome in ICU patients[9,11], and we accordingly found a 2.2 times higher proportion of infection, a 5.5 times higher risk of septic shock, a 3.7 times higher proportion of bacteraemia and a 6.9 times higher 28-day mortality among SIRS patients than among non-SIRS patients.

We found SIRS status to be highly correlated with 28-day mortality, which is in contrast to a previous study of patients from an emergency ward with suspected infection [8]. Interestingly, we found that the high 28-day mortality among SIRS patients was largely attributable to patients without documented infection on arrival (13/15 deaths), which means that SIRS among patients without infection is a bad prognostic factor, reflecting the fact that SIRS is a general expression of the degree of acute physiological disturbance which the patient is suffering [17]. In the present study, most of the deaths among patients with SIRS but no infection occurred among patients with malignant conditions, which highlights the prognostic importance of pre-existing conditions. Similarly, a previous study found that SIRS patients without infection had more comorbidity and a higher mortality than patients without SIRS [9].

The 28-day mortality was 1/42 (2%) among septic patients, 1/20 (5%) among patients with severe sepsis, and 0/5 (0%) among patients with septic shock. Given the low numbers, the results must be interpreted with caution, but the observed mortality is lower than in most other studies of patients with sepsis and septic shock [3-7,9,10]. A probable explanation is a difference in patient selection and inclusion criteria. For example, we included patients with infection documented according to predefined rules. A different definition of documented or suspected infections would change the reported mortality. In the present study we had a systematic selection of patients with suspected heart disease, confirmed diabetes and other chronic disorders who were systematically admitted to other wards, and we may have included patients with less comorbidity than in other studies. Our patients were identified by symptoms on arrival and signs of community-acquired infection, whereas most other studies include patients from intensive care units or they identified patients by discharge diagnosis. These studies include patients with community-acquired as well as nosocomial infections. As in the present study, two studies of emergency ward patients with suspected infection showed an in-hospital mortality of 1% for uncomplicated sepsis and 4-9% for severe sepsis [8,15].

As SIRS symptoms on arrival are related to infection as well as 28-day mortality, it might be useful to make a systematic registration of this among acute medical patients. However, 38% of the infected patients did not have SIRS on arrival, and they would be missed if SIRS were used as the only way to identify infected patients. If the main purpose was to identify patients with a high risk of mortality, the question is whether a systematic SIRS registration of acute medical patients offers more information and gives better guidance to the clinician than he or she had in advance.

From a clinical epidemiological point of view, a systematic registration of SIRS status in a patient arriving at a medical emergency ward may provide improved information for decision making in management of the patient. The symptoms provide information to the clinical doctor on the degree to which he or she can expect infection in a patient presenting with SIRS, but also provides information of an expected high 28-day mortality. SIRS symptoms provide information on a patient with a highly activated immune response due either to infections or to other conditions, and a systematic registration of the symptoms might serve to further sharpen attention among the staff in medical emergency wards. SIRS patients in a medical emergency ward are a very diverse group. We believe a better understanding of the different patient subcategories can benefit future selection of patients for specific therapies. Whether or not a systematic registration of SIRS status improves decision making and treatment in the medical emergency ward is still unknown, but it would be possible to test this with, for example, a randomised design.

## In conclusion

In acutely hospitalised medical patients, the prevalence of SIRS is high (35%). SIRS is only moderately related to infection on arrival, but is highly related to 28-day mortality.

## Competing interests

The authors declare that they have no competing interests.

## Authors' contributions

PC contributed to the design of the study, obtained data, made the analysis, interpreted the data and wrote the first draft. MS and AL contributed to the design of the study and the interpretation of the data and made a critical revision of the manuscript. All authors have read and approved the final manuscript.
